# Stem Cells for Augmenting Tendon Repair

**DOI:** 10.1155/2012/291431

**Published:** 2011-11-29

**Authors:** Lawrence V. Gulotta, Salma Chaudhury, Daniel Wiznia

**Affiliations:** Sports Medicine and Shoulder Service, Hospital for Special Surgery, 535 E 70th Street, New York, NY 10021, USA

## Abstract

Tendon healing is fraught with complications such as reruptures and adhesion formation due to the formation of scar tissue at the injury site as opposed to the regeneration of native tissue. Stem cells are an attractive option in developing cell-based therapies to improve tendon healing. However, several questions remain to be answered before stem cells can be used clinically. Specifically, the type of stem cell, the amount of cells, and the proper combination of growth factors or mechanical stimuli to induce differentiation all remain to be seen. This paper outlines the current literature on the use of stem cells for tendon augmentation.

## 1. Introduction

Tendons function to transmit force from muscle to bone, with the ultimate effect of actuating motion. Tendon tissue has been designed to endure large tensile loads. When tendons are injured, normal tendon histology is not restored and, therefore, neither is function. Tendons heal with an intervening layer of scar tissue. This scar tissue has material properties that are inferior to native tendon. This makes surgical repairs of torn tendons prone to failure. It also makes them susceptible to adhesion formation due to excess fibrous tissue formation. Therapies that can augment regeneration of normal tendon and limit the amount of scar tissue that is formed in response to injury may improve clinical outcomes.

Stem cells have great promise in enhancing the biologic healing process since they provide a self-renewing population of pluripotent cells. However, several questions still remain before stem cells can be used clinically for augmenting tendon healing. Specifically, the type of stem cell, the amount of cells, the combination of growth factors and mechanical stimuli, and the ideal delivery vehicle all still need to be determined. The purpose of this paper is to outline the current research on developing a clinical stem cell therapy for the augmentation of tendon healing.

## 2. Types of Stem Cells

Stem cells are defined as a population of cells that can self-renew through symmetrical mitotic division, form daughter cell lines, and generate a broad range of tissue lineages through terminal differentiation [1, 2]. Stem cells can be derived from a number of sources, and thus different stem cell categories exist. These categories include embryonic, peri-natal (obtained from the umbilical cord or from amniotic tissue), somatic adult, or induced pluripotent stem cells (iPSCs). While there is some overlap, the most common categories of adult stem cells are mesenchymal stem cells (MSCs) and hematopoietic stem cells which are both defined based upon specific stem cell surface markers [3]. iPSCs are not adult stem cells in origin but mature adult cells that are modified resulting in cell pluripotency and the characteristics of embryonic cells [4, 5]. This exciting technique holds great promise for the future, but there has been very little investigation of their usefulness for orthopaedic interventions. In view of ethical concerns and current regulatory issues associated with embryonic or perinatal stem cells, orthopaedic stem cell research has predominantly focused upon MSCs.

**Figure 1:**
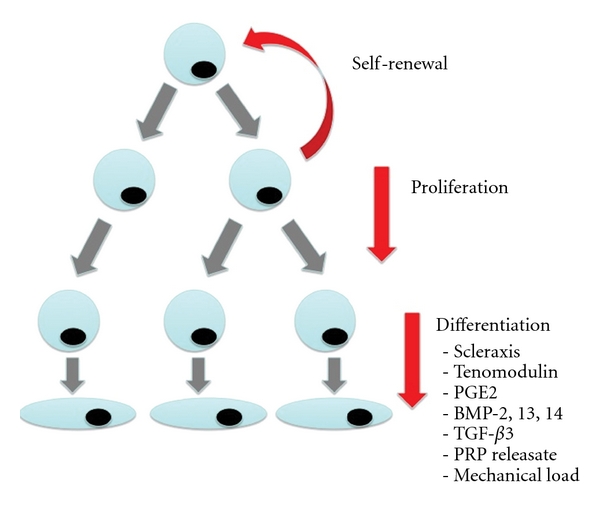
Pathway of mesenchymal stem cell differentiation into tenocytes.

MSCs (also referred to as mesenchymal stromal cells) are defined by their ability to self-renew and their multipotentiality [6]. MSCs are defined by three characteristics: (i) an ability to adhere to plastic, (ii) presentation of stem cell specific antigens, and (iii) the potential to form multipotent mesenchymal cells which can differentiate into a number of cell lines interesting to musculoskeletal medicine such as osteocytes, chondrocytes, and adipocytes [7–9]. No stem-cell-specific marker has been isolated to date, although numerous stem-cell-associated positive and negative markers have been identified. Stem cell associated positive markers include CD 31, 34, 40, 49c, 53, 74, 90, 106, 133, 144, and 163, as well as cKit and Slams [10–13]. Negative stem cell markers indicate other cell lineages such as hematopoietic and endothelial cells and include CD 14, 31, 34, and 45 [14–17]. Induction of MSCs into specific cell lineages such as tenocytes is determined by culturing processes as well as growth and media conditions (Figure 1).

Bi et al. identified a tendon progenitor stem cell (TPSC) population in both mice and humans [14]. A greater propensity of TPSC was identified in “niches,” or specialized tendon microenvironments, that contain an array of growth factors such as biglycan and fibromodulin. TPSC can be differentiated from tenocytes by the presence of stem cell markers such as Oct-4, tenomodulin, and SSEA-4 [16]. Multidifferentiation potential is maintained within the TPSC population as they can differentiate into tenocytes, chondrocytes, osteocytes, and adipocytes. Prostaglandin E_2_ (PGE_2_), BMP-2, BMP-12 and -13 TGF-*β*_3_, and platelet-rich plasma releasate are proposed to be important mediators for promoting stem cell differentiation into tendon tissue as opposed to adipocyte and osteocyte formation [15, 18]. Tendon progenitor stem cells decrease with age, which may contribute to the age-related reduction in tendon repair seen in rotator cuff tears [19]. Increasing the pool of tendon stem may stimulate increased tendon healing with tendon regeneration rather than reactive scar formation.

MSCs have been isolated from a number of different tissue sources. The most common sources of MSCs for musculoskeletal applications are bone marrow and adipose tissue due to their accessibility and the ability to obtain large numbers of viable cells [20]. Bone-marrow-derived MSCs have a greater ability to differentiate into chondrocytes and osteocytes compared to adipose-derived MSCs [21, 22], although the latter provides greater ease of access and decreased donor site morbidity [23]. However, human bone marrow consists of very low yields of MSCs, accounting for only 0.001–0.01% of total nucleated cells [8]. Less common sources of MSCs include tendon, muscle, synovium, cartilage, skin, peripheral blood, periodontal tissue, hair follicles, and scalp tissue [18, 24–26]. While MSCs are relatively easy to harvest, there is concern about their ability to efficiently differentiate into tendon. Research into embryonic, perinatal, and iPSCs is fastly emerging, though their usefulness for tendon augmentation remains to be seen.

## 3. Untreated Stem Cells

As MSCs possess the potential to differentiate into tenocytes, MSC application to torn tendons is proposed to recapitulate tendon development signals and improve tendon healing capabilities. MSC-mediated tendon regeneration has been studied in numerous animal tendon models.

The addition of MSCs to semitendinosus tendons in bone tunnels during anterior cruciate ligament (ACL) reconstructions promoted improved healing with higher failure loads and stiffness after 8 weeks [27]. Healing occurred through fibrocartilagenous tissue that resembled the native tendon-to-bone insertion site rather than the scar tissue typically seen following injury. Lim et al. also delivered MSCs in a fibrin glue carrier to tendon grafts in a rabbit ACL reconstruction model [28]. At 2 weeks, large numbers of immature fibrocartilage cells were noted at the tendon enthesis, and, by 8 weeks, the enthesis resembled a normal ACL insertion with a mature fibrocartilagenous zone as well as improved load to failure and stiffness properties. In a similar study by Soon et al., synovium-derived MSCs were added to rabbit ACL repairs [20]. Compared to the control group without cells, MCS-coated grafts produced a fibrocartilagenous zone resembling a normal ACL insertion. While the MSC-treated group produced significantly higher load to failures, it also had lower stiffness and Young's modulus values suggesting that MSCs mediated a more physiologic regenerative response.

Awad et al. demonstrated that bone-marrow-derived stem cells delivered to a patellar tendon wound site resulted in improved mechanical and some histological properties [24]. The addition of MSCs resulted in increased maximum stress, modulus, and strain in addition to improved mature collagen fibers and cells. However, there was no significant improvement in microstructure compared to the control group, and ectopic bone formation was also noted.

Another study used a rabbit Achilles model with a 1 cm tendon defect that was augmented with MSCs suspended in collagen gel that had been contracted onto pretensioned sutures [29]. MSC treatment resulted in superior load properties, collagen fiber alignment, and cross-sectional area at up to 12 weeks. Despite the increase in tissue volume, the regenerated tissue was composed of fibroblast-like cells rather than tenocytes. A study by Chong et al. reported an early improvement in mechanical and histologic properties at three, but not six weeks following MSC delivery to a rabbit Achilles tendon model [25]. Labeled bone-marrow-derived MSCs were noted to be viable and localized to the intratendinous region at six weeks, before displaying a more diffuse spread. This suggested that any MSC-mediated acceleration in healing occurs during the early phase of healing. Synovium-derived MSCs have also been shown to accelerate early remodelling of Achilles tendons by stimulating increased collagen fiber production at 1 week and fibers resembling Sharpey's fibers between the tendon and bone at 2 weeks [26].

A number of animal studies suggest that simply delivering stem cells alone not appear to improve tendon healing. Gulotta et al. showed that the application of bone-marrow-derived MSCs alone was insufficient to improve rotator cuff healing in a rat model [30]. The effectiveness of MSCs may be determined by achieving targeted and sustained stem cell delivery to the site of tendon defects, as well as through appropriate modulation of growth factors, cytokines, mechanical environment, and cell concentration.

A few studies have tried to address the effect of different stem cell densities. MSCs were seeded onto collagen matrices and contracted onto sutures at different cells densities of 1, 4, and 8 million cells/mL prior to implantation into rabbit patellar tendon defects [31]. The repairs with MSC collagen composites demonstrated significantly higher maximum stresses and moduli, as well as faster healing than repairs without MSCs. The mechanical improvements did not correlate with improved histological appearance, although greater tissue volume was noted. Interestingly, no difference was detected between the different cell densities, suggesting that higher MSC concentrations do not necessarily translate to improved healing. Of concern was the fact that 28% of the MSC-mediated repairs developed ectopic bone formation at the repair site. A similar study by Juncosa-Melvin et al. investigated the effect of seeding different MSC densities onto tissue-engineered constructs to repair patellar defects in rabbits [32]. No differences in mechanical properties were detected between the two cell densities or between patellar tendon defects treated with or without cells.

Some studies have tried to address whether stem cells mediate a superior effect on tendon healing compared to alternative types of differentiated cells. Hankemeier et al. used a patellar tendon defect model to compare the effects of stem cells and human fibroblasts that were both delivered using a fibrin matrix [33]. Stem cells were found to produce better tendon healing with a significantly increased mean collagen fibril diameter and area covered by collagen fibrils after 10 but not 20 days, in addition to increased collagen 1 and 3 mRNA expression at 20 days. However, the different cell types did not produce a difference in mechanical properties, as measured by ultimate stress.

## 4. Growth and Differentiation Factors and Stem Cells

While most animal studies have shown encouraging results with the simple application of untreated stem cells, a growing consensus exists that optimal therapy will include treatment of these cells with growth factors. Many studies have demonstrated that mesenchymal stem cells differentiate in response to bone morphogenetic proteins, transforming growth factors and fibroblast growth factor [34].

Gulotta et al. have studied the effect of various growth or differentiation factors and stem cells on tendon healing in a rat model of rotator cuff repair [35–37]. They did not find any improvement in histologic and biomechanical outcomes when MSCs were transduced with BMP-13 when compared to untransduced MSC controls [36]. However, they did find improved healing parameters when MSCs were transduced with two genes present during tendon development during embryogenesis, scleraxis [35], and membrane type 1- matrix metalloproteinase (MT1-MMP) [38]. When MSCs transduced with either scleraxis or MT1-MMP were added to the healing site, improved histology scores and biomechanical strength were seen as early as 4 weeks following repair. This led the authors to conclude that certain growth or differentiation factors will be necessary, in addition to MSCs, in order to optimize their effectiveness.

In another study, investigating the role of bone morphogenetic proteins, Rui et al. modeled calcifying tendinopathy with an in vitro model that applied uniaxial cyclic loading to tendon-derived stem cells grown on plates coated with collagen type I [39]. Increased repetitive tensile loads led to increased expression of BMP-2 as well as increased cell alignment along the direction of externally applied tensile forces. In addition, BMP-2, when added to tendon derived stem cells, was demonstrated to promote osteogenic differentiation and led to ectopic calcification.

While growth factor therapy in addition to MSC seems promising, the question remains as to what factor yields the best results. The various possible combinations of factors are daunting, which has led researchers to pursue platelet rich plasma (PRP) as a way of delivering multiple factors in a single therapeutic agent. PRP has been shown to influence the behavior of stem cells. Using tendon stem cells derived from rabbit patellar tendons, Zhang and Wang demonstrated that increased PRP releasate caused tendon stem cells to become larger and elongated [18]. PRP releasate increased tendon stem cell proliferation, induced tendon stem cell differentiation into tenocytes, and increased protein expression and collagen type I and type III production.

## 5. Mechanical Load and Stem Cells

In addition to growth factors, altering the mechanical environment is another way to induce stem cell differentiation into tenocytes. The mechanical loading of stem cells appears to guide differentiation. Sharma and Snedeker combined mechanical and molecular cues by culturing bone marrow stromal stem cells on a hydrogel matrix with a gradient of mechanical compliance as well as gradients of the ligands fibronectin and collagen type I [40]. These cells differentiated towards osteogenic precursors on fibronectin and tenogenic precursors on the collagen substrate. In addition, osteogenic differentiation increased on stiffer fibronectin substrates and decreased on collagen substrates. Tenogenic differentiation was only observed on collagen substrates within a narrow range of stiffness. Yin et al. further demonstrated that cells sense matrix topography and that this information effects gene expression and differentiation [41]. Human tendon progenitor cells were seeded on aligned or randomly oriented poly nanofibers. The expression of tendon-specific genes, such as integrin alpha 1, alpha 5, and beta 1 subunits and myosin B was found to be higher in cells growing on aligned nanofibers than those on randomly oriented nanofibers. In addition, randomly oriented nanofibers induced osteogenesis, as demonstrated by increased alkaline phosphatase activity, while aligned scaffolding hindered osteogenesis. This work demonstrates that nanotopography, with an aligned organization, induces tendon stem cells to form tendon-like tissue in vivo.

As work progresses on the development of biomimetic scaffolds for tendon tissue, the mechanical loading of tissue has also been demonstrated to influence stem cell differentiation [42]. Zhang and Wang recently demonstrated, in an in vitro model, how the magnitude of mechanical loading influences the route of differentiation of tendon stem cells [43]. Low mechanical stretching at 4% strain directed tendon stem cells into tenocytes, while stretching at 8% directed stem cells into adipogenic, chondrogenic, and osteogenic lineages. Zhang and Wang also reported, in a live animal rat study, that treadmill running doubled proliferation rates of tendon stem cells in both the patella and Achilles tendon as well as cellular production of collagen [44]. In addition, the lab demonstrated that stem cells isolated from the treadmill group produced more collagen when cultured with tenocytes than stem cells isolated from the cage control group. This indicates that the proper mechanical loading conditions increase the proliferation of tendon stem cells as well as cellular production of collagen.

While mechanical signals have been shown to affect mesenchymal stem cells fate, the characteristics of an effective loading regimen for tendon-to-bone healing have yet to be fully determined. Sen et al., using an in vitro model in which mesenchymal stem cells underwent two twenty-minute episodes of low intensity vibration or high magnitude strain, demonstrated that adipogenesis was suppressed and osteogenesis was amplified when there was at least a one-hour refractory period between bouts [45]. The group further showed that the effect was enhanced with increasing lengths of the refractory periods. This work shows that the scheduling of loading events is at least as important as the magnitude of the loads.

## 6. Clinical Applications of Stem Cells

A number of clinical applications for augmenting tissue healing with stem cells in humans have been reported. MSCs have been used for human craniofacial tissue regeneration with varying reports of success [46]. Orthopaedic stem cell studies in humans have predominantly focused upon enhancing bone healing, particularly in spine, foot and ankle, and fracture surgery [47, 48]. Centeno et al. treated 227 patients with autologous MSCs that were cultured and then injected into peripheral joints (*n* = 213) or intervertebral discs (*n* = 13) [49]. Patients underwent disease surveillance for an average of 10.6 months, and no malignant transformations were reported. One patient was diagnosed with cancer which the authors believe was “certainly unrelated” to the MSC therapy. Seven patients had complications related to the injection, and 3 possible stem-cell-related complications were reported. Forty-five of the patients had serial MRIs for up to 2 years, and none of the patients showed any evidence of tumor formation, suggesting that MSC therapy is a relatively safe and well-tolerated procedure. However, the few clinical stem cell studies have only reported short-term outcomes and ideally longer-term followup is required to determine safety.

Despite the promising MSC-mediated effects on tendon healing noted in a number of animal studies, there are no clinical studies examining the efficacy and safety of stem cells on human tendon repair. However, other cellular therapies have been shown to clinically improve tendon healing. Clarke at al. applied skin-derived tenocyte-like cells to 33 patients with patellar tendinopathy and compared it to 27 patients who were treated with plasma [50]. The group receiving stem cell treatment noted a significant improvement in Victorian Institute of Sport Assessment (VISA) scores. While both the cell and plasma groups resulted in an improvement in tendon hypoechogenicity on ultrasound and tear size, only the cell group showed a significant decrease in tendon thickness. A pilot study of 12 patients with refractory elbow epicondylitis showed a significant improvement in the patient-rated tennis elbow evaluation scale and ultrasound tendon appearance [51].

**Figure 2:**
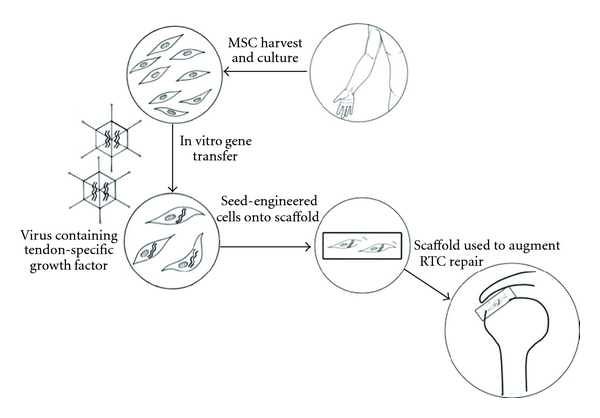
One potential clinical application of stem cell technology for tendon repair.

A number of issues regarding the clinical application of stem cells still need to be addressed. The method of stem cell aspiration may affect the viability and function of the cells. Stem cell therapies typically require in vitro culturing and expansion of cells to obtain sufficient numbers of viable cells prior to reimplantation (Figure 2). This raises a number of ethical and regulatory concerns. A one-step approach is increasingly advocated, wherein a sufficient number of stem cells are harvested intraoperatively, concentrated, and then reimplanted within the same procedure. This technique is considered an intraoperative procedure using autologous tissue, and as such does not require Food and Drug Authority (FDA) approval. Hernigou et al. have successfully used this technique to treat patients with femoral osteonecrosis and fracture nonunions [52, 53]. A number of commercial bone marrow aspirate concentration systems are available which can concentrate cells within an hour. 

The source of stem cells may help to determine stem cell effectiveness. Locally derived stem cells may most likely to be effective in promoting tendon regeneration, particularly in view of the importance of tendon “niches” in tenocyte differentiation humans [14]. Progenitor cells with osteogenic potential were aspirated from the proximal humerus of 23 patients during arthroscopic rotator cuff repairs [54]. No adverse effects of stem cell aspiration were detected on clinical outcomes, although the aspirated cells were not reinjected into the patients. The authors proposed that the proximal humerus is an efficient and safe site for harvesting progenitor cells during rotator cuff repairs. Using a reamer-irrigation-aspirator (RIA) during reaming of long bones was shown to permit isolation of MSCs which were comparable to iliac crest bone-marrow-derived cells in terms of differentiation potential and daughter line phenotypes [55]. However, the RIA produced greater numbers of colony forming units and total cell numbers compared to collecting cells from the iliac crest.

Effective translation of promising stem-cell-mediated tendon healing in animals has not yet been proven in the clinical setting. A number of concerns exist about MSC use within humans, such as tumor-like growth of MSCs and modulation of the immune system. Regardless of the source of MSCs, there is a risk of differentiation into undesirable lineages which could result in ectopic tissue formation and calcium deposition. Systemic injections of allogeneic MSCs have been shown to disseminate to a number of organs in baboons [56]. Some of the potential concerns are associated with the culturing process of MSCs and include the risk of genetic alterations, phenotypic drift, as well as transmission of zoonotic infections from the use of fetal bovine serum during culturing.

## 7. Conclusions

Stem cells are an attractive option for the augmentation of tendon repairs. Embryonic stem cells appear to offer the best differentiation potential, but their use is controversial, and no studies have evaluated their clinical usefulness. Several studies have evaluated the use of MSCs with some success, but profound results have been lacking when these cells are applied untreated. Molecular cues, such as the addition of BMP's, MT1-MMP, and scleraxis, and mechanical cues such as changes in strain and nanotopography, have shown promising results in their ability to drive MSCs into tenocytes differentiation. Utilizing this knowledge to develop a clinically useful therapy has yet to be accomplished, but our understanding of stem cell biology continues to expand with the hope of one day finding one.
